# Surface-Stress-Induced Lattice Distortion in Nanolamellar Cementite

**DOI:** 10.3390/ma19163431

**Published:** 2026-08-13

**Authors:** Marek Gocnik, Maximilian Graf, Peter Kunnas, Anna Sophie Jelinek, Daniel Marian Ogris, Ronald Schnitzer, Jozef Keckes

**Affiliations:** 1 Christian Doppler Laboratory for Knowledge-Based Design of Advanced Steels, Department of Materials Science, Montanuniversität Leoben, 8700 Leoben, Austria; maximilian.graf@unileoben.ac.at (M.G.); ronald.schnitzer@unileoben.ac.at (R.S.); jozef.keckes@unileoben.ac.at (J.K.); 2 Erich Schmidt Institute of Materials Science, Austrian Academy of Sciences, 8700 Leoben, Austria; peter.kunnas@oeaw.ac.at; 3 Department of Material Science, Montanuniversität Leoben, 8700 Leoben, Austria; anna.jelinek@unileoben.ac.at; 4 voestalpine Forschungsservicegesellshaft Donawitz GmbH, Kerpelystrasse 199, 8700 Leoben, Austria

**Keywords:** steels, cementite, nanocrystalline microstructure, synchrotron radiation, surface stress

## Abstract

Cementite morphology is known to govern the mechanical performance of steels, yet its influence on actual crystal structure, like unit cell parameters, is not well quantified. Here, we show that cementite morphology-dependent surface stresses generate measurable lattice distortion in nanocrystalline cementite. Lamellar and spheroidized cementite powders were extracted from a Cr-alloyed hypereutectoid steel and characterized by high-energy synchrotron X-ray diffraction combined with double-Voigt profile modeling. Despite a higher Cr content in spheroidized cementite, lamellar cementite exhibits systematically larger lattice parameters, corresponding to an average volumetric lattice strain of 6.3 ± 2.2 × 10^−4^. This counter-intuitive expansion is rationalized by a continuum mechanics framework that incorporates the orthorhombic elastic anisotropy of cementite and particle morphology. The model predicts the sign and magnitude of morphology-induced lattice strain, demonstrating that surface-stress effects constitute a systematic bias in diffraction-derived lattice parameters of nanostructured carbides.

## 1. Introduction

Steel derives its mechanical versatility from diverse microstructures and phase compositions [1,2,3,4], among which cementite morphology plays a central role in governing dislocation motion, strain partitioning and damage evolution [5,6,7,8]. In the cooperative eutectoid transformation, ferrite and cementite grow from a common transformation front to form a pearlitic microstructure with lamellar cementite, providing high strength and wear resistance and enabling widespread use in rails, cutting tools, and high-strength wires [9,10,11]. In contrast, the divorced eutectoid transformation [12] produces a microstructure consisting of spheroidized cementite precipitates dispersed in a ferrite matrix, which is preferred when enhanced ductility, fracture toughness and machinability are required [13,14,15,16,17]. While this morphology–property relationship is well established, the extent to which thermal treatment and the resulting cementite morphology affects the lattice parameters of cementite remains largely unexplored. The high surface-to-volume ratio of cementite lamellae implies that surface energy and the associated near-surface stresses can modify lattice parameters of cementite. In nanocrystalline materials, like lamellar cementite, these surface-induced stresses may bias diffraction-derived lattice parameters and strain states even in the absence of external load. Diffraction and first principles studies on crystallographic properties of nanolamellar cementite have primarily addressed the effects of thermal treatment and composition—such as lattice expansion due to Cr substitution—without specifically isolating the contributions arising from morphology-dependent surface effects [18,19]. Consequently, both the magnitude and sign of morphology-driven lattice strain in cementite remain poorly quantified, despite its importance for interpreting residual stresses and phase stability in steels. In this work, we quantify morphology-dependent mean lattice strain in cementite by combining high-energy synchrotron powder X-ray diffraction (HE-XRD) with diffraction-profile modeling. A Cr-alloyed hypereutectoid steel was subjected to a conventional eutectoid heat treatment and a divorced eutectoid route to generate lamellar and spheroidized cementite, respectively, followed by selective electrochemical dissolution of ferrite to isolate phase-pure cementite powders. The experimentally observed differences between the lattice parameters and diffraction peak broadening are interpreted using an analytical, surface-energy continuum mechanics-based model that accounts for the elastic anisotropy of cementite’s orthorhombic crystal structure and the particular morphology of the cementite particles. The proposed framework provides a general approach for understanding how nanoscale morphology influences lattice strain through surface-induced stresses. Such effects are particularly relevant in nanostructured materials with diverse morphologies, where variations in surface-to-volume ratio and geometric constraints can generate distinct stress states that influence diffraction-derived lattice parameters. By establishing a direct relationship between particle morphology and lattice strain, this approach enables the contribution of morphology-dependent surface effects to be distinguished from other factors, such as compositional and thermal history. Using spheroidized cementite as an effectively unstrained reference state, the proposed model reproduces the experimentally observed expansion of lattice parameters in lamellar cementite and captures the correct order of magnitude of the morphology-induced strain. This demonstrates the significance of morphology-dependent surface stress in defining the lattice state of nanostructured materials at large.

## 2. Materials and Methods

**Figure 1:**
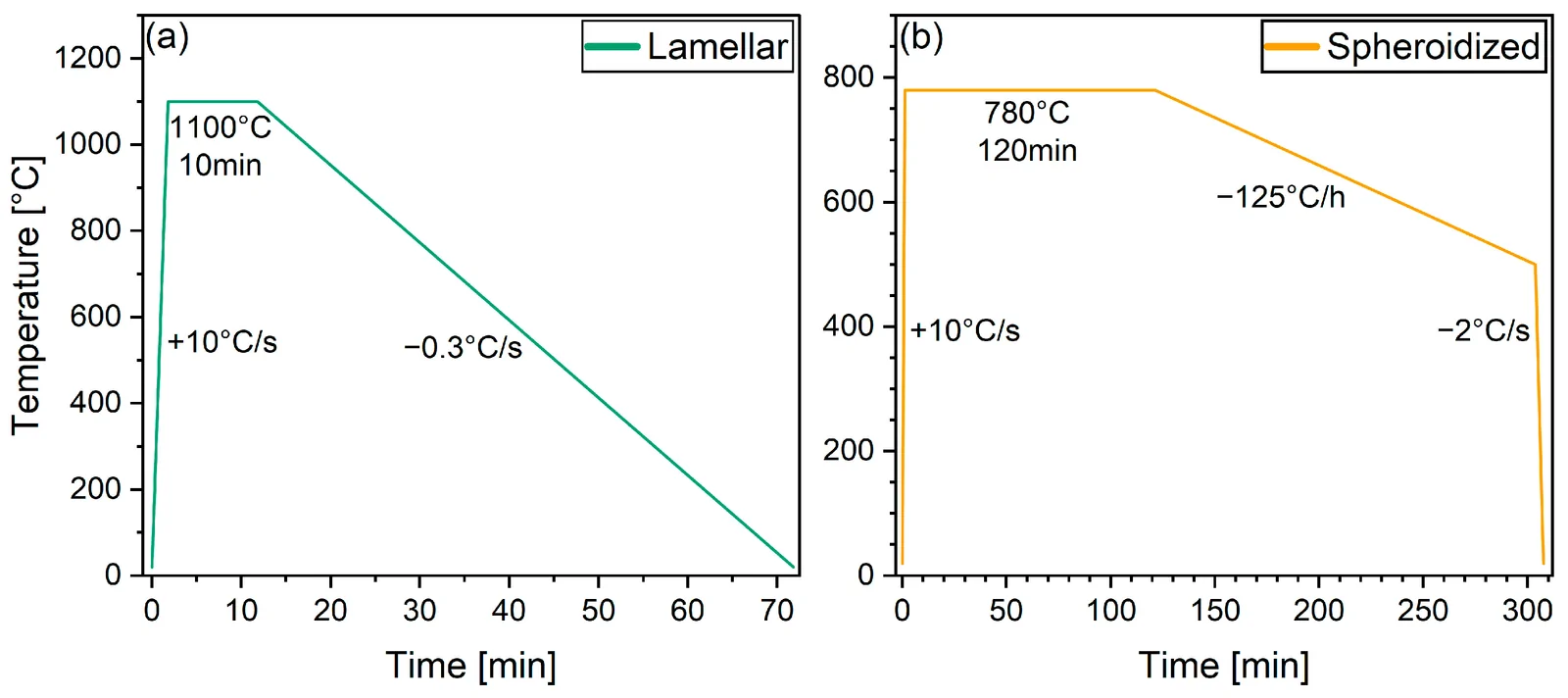
Applied heat-treatments to obtain (**a**) pearlitic microstructure with lamellar cementite and (**b**) divorced eutectoid microstructure containing spheroidized cementite particles.

Two samples of a Cr-alloyed hypereutectoid steel with identical nominal composition of approximately 1 wt.% C and 1.5 wt.% Cr were subjected to (i) a conventional austenitization heat treatment to generate a pearlitic morphology with lamellar cementite and (ii) a divorced eutectoid thermal cycle to obtain spheroidized cementite precipitates dispersed in a ferrite matrix. Both heat treatments were performed in a Bähl DIL805 A/D quenching dilatometer (TA Instruments, New Castle, DE, USA). The conventional heat treatment involved complete austenitization at 1100 °C for 10 min, followed by controlled cooling at a rate of 0.3 °C/s. In contrast, the divorced eutectoid route employed partial austenitization in the γ-Fe + Fe_3_C field at 780 °C for 120 min, followed by a slow cooling at a rate of 125 °C/h (0.03 °C/s). After reaching the temperature of 500 °C the sample was cooled to room temperature at a rate of 2 °C/s. Applied heat treatments are presented in Figure 1a and Figure 1b for the conventional and divorced eutectoid, respectively.

Final microstructures were characterized by a Tescan Magna scanning electron microscope (SEM) after electrochemical polishing (TESCAN, Brno, Czech Republic). Bright-field transmission electron microscopy (TEM), combined with energy-dispersive X-ray spectroscopy (EDS), was performed using a JEOL JEM-2200FS electron microscope equipped (JEOL Ltd., Tokyo, Japan) with an Oxford AZtecEnergy UltimMax detector (Oxford Instruments NanoAnalysis, High Wycombe, UK). TEM samples were prepared using a Xe-plasma focused ion beam (pFIB) with a Thermo Fisher Helios 5 CXe (Thermo Fisher Scientific, Waltham, MA, USA), following a standard lift-out and thinning procedure that resulted in a lamella thickness of approximately 100 nm. Tensile specimens were fabricated according to the Ref. [20] standard (DIN 50125-M10) by wire electrical discharge machining and tensile tests were conducted at room temperature on a AGG 50 kN (Hegewald&Peschke, Nossen, Germany) universal testing machine at a controlled strain rate of 1 × 10^−3^ s^−1^. Pure phase cementite powders were extracted from separately heat-treated dilatometer samples via electrolytic dissolution in a methanol—10 wt.% HCl electrolyte at 5 V and 60 °C, then washed, dried and loaded into capillaries for HE-XRD analysis at the ID22 beamline of ESRF in Grenoble, France [21]. XRD measurements were conducted at room temperature in transmission geometry, using a monochromatic X-ray beam with an energy of 35 keV (0.35431 Å) and a beam size of 1 × 1 mm^2^. Recorded powder diffractograms were analyzed by Rietveld refinement [22], incorporating double-Voigt size-strain models following Ectors et al. [23] and Stephens [24], as implemented in TOPAS v7 (version: 7.13, Bruker AXS GmbH, Karlsruhe, Germany) [25,26]. The double-Voigt approach was used to describe the morphology-dependent diffraction peak broadening and to separate and quantify the coherent domain size and microstrain peak-broadening contributions. The crystallite shape was parametrized using geometric models: spheroidized cementite was represented by a triaxial ellipsoid with equal principal radii (spherical approximation), whereas lamellar cementite was described using a cuboid model (lamellar approximation) with three independent size parameters. Raw XRD data and TOPAS input files are available through Mendeley Data at 10.17632/7xcrjd6dxx.1.

## 3. Results and Discussion

**Figure 2:**
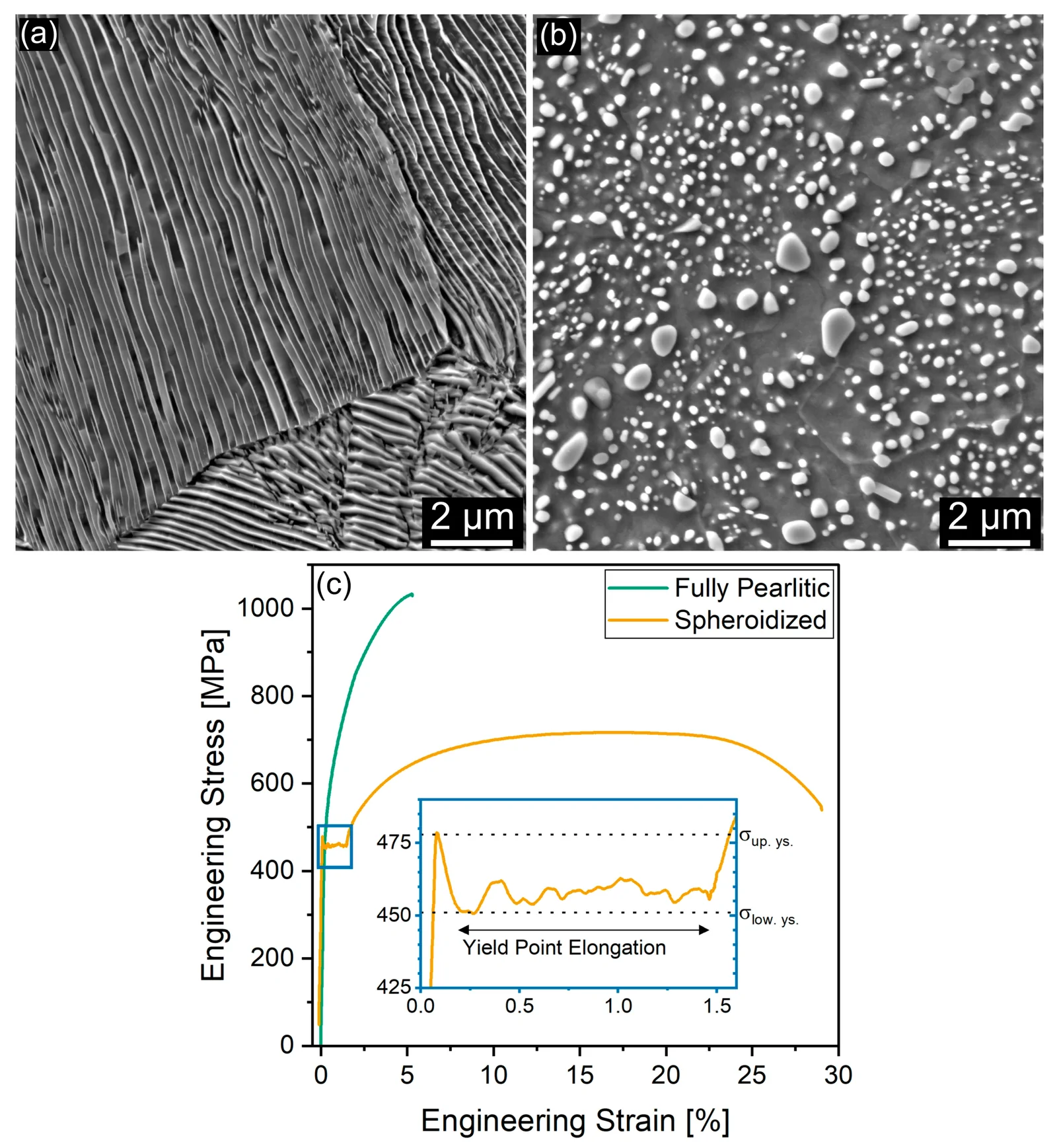
Representative SEM cross-section micrographs of investigated samples following the heat treatments presented in Figure 1 are shown for the (**a**) fully pearlitic and (**b**) divorced eutectoid (spheroidized) microstructure. In (**c**), representative engineering stress–strain curves obtained from uniaxial tensile loading are shown.

The SEM micrographs, shown in Figure 2a,b, confirmed a formation of a fully pearlitic microstructure [9], characteristic for hypereutectoid steels, whereas the alternative route yielded a divorced eutectoid microstructure with spheroidized cementite particles dispersed within a ferritic matrix.

Representative SEM micrographs of the spheroidized cementite were analyzed using an in-house image recognition routine to quantify cementite precipitate size and morphology. The resulting equivalent circle diameter (ECD) and aspect-ratio distributions, compiled from more than 3000 cementite particles, are presented in Figure S1. The analysis yielded a mean particle size of 220 ± 89 nm. The aspect-ratio distribution indicates a predominantly equiaxed morphology, with 75% of the particles exhibiting an aspect ratio below 1.5.

Representative engineering stress–strain curves, presented in Figure 2c, underscore a strong microstructural dependence of tensile properties: with continuous yielding in the pearlitic condition and pronounced strain hardening (R_p0.2_ = 566 ± 1 MPa, ultimate tensile strength (UTS) = 1036 ± 2 MPa) and fracture at 4.8 ± 0.4% strain with no post-uniform deformation. In contrast, the spheroidized condition showed reduced strength (UTS = 713 ± 2 MPa) but markedly higher ductility with yield-point behavior and fracture at 29.3 ± 0.2% elongation. The markedly higher ductility of the divorced eutectoid microstructure in combination with post-uniform deformation underpins its utility in steel-wire production and other forming-intensive applications. Furthermore, the observed discontinuous yielding is indicative of Lüders-band formation and propagation, i.e., yield-point behavior governed by abrupt dislocation unpinning followed by band-controlled plastic flow at an approximately constant stress [27,28]. Recorded engineering stress–strain curves for all specimens and determined mechanical properties are presented in Figure S2 and Tables S1 and S2.

**Figure 3:**
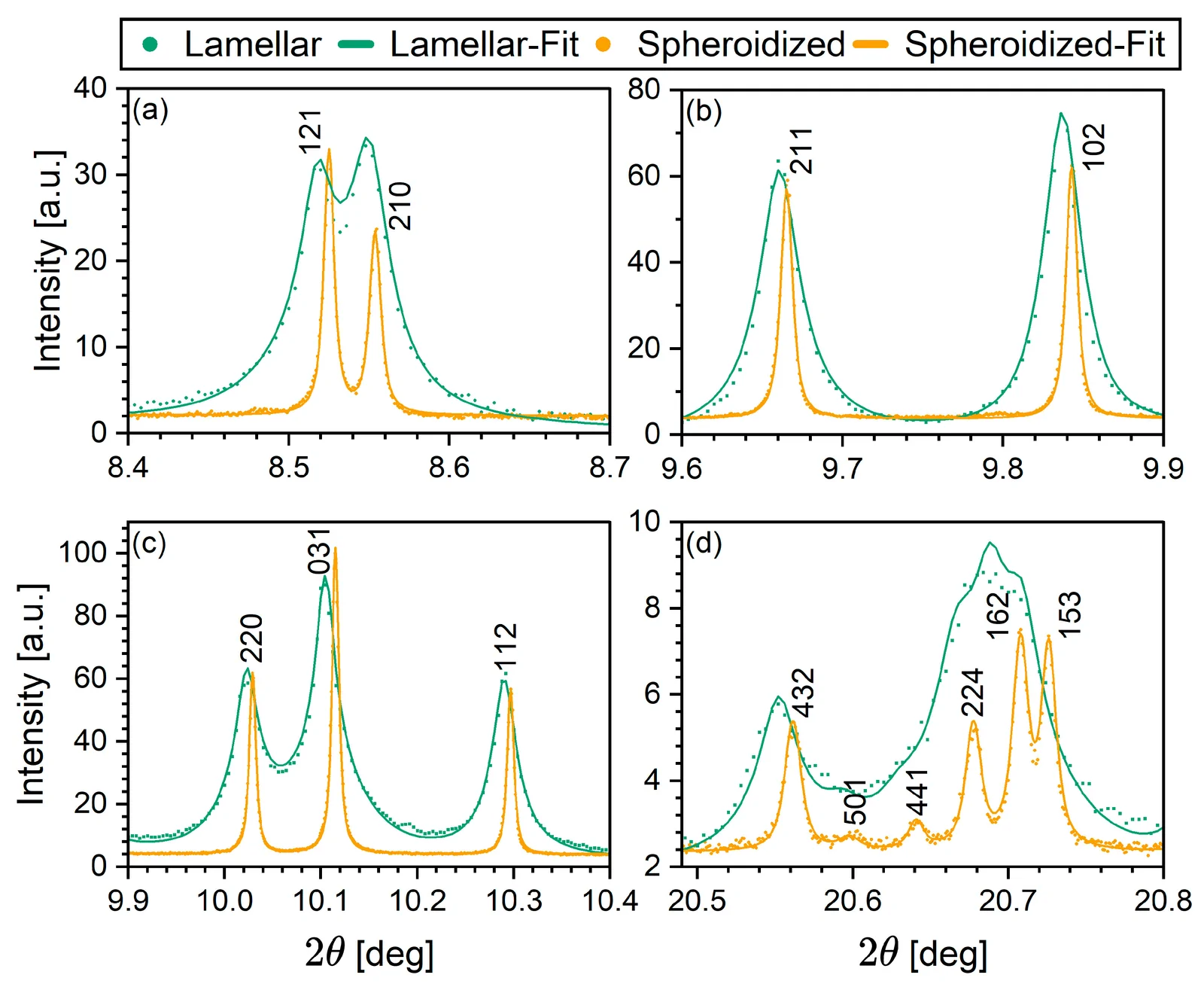
In (**a**–**d**) selected 2*θ* regions of recorded powder diffractograms, shown in Figures S3 and S4, for the lamellar and the spherical cementite morphologies are shown, respectively. Discrete points represent experimentally recorded diffraction data, and continuous lines represent modeled diffraction profiles. Additionally, Miller indices of identifiable reflections are also indicated.

Of the two powder XRD datasets, each spanning a Bragg’s angle range 2*θ* of 5–30 degrees and containing more than 150 Fe_3_C reflections, shown in Figures S3 and S4, representative regions are presented in Figure 3.

Considering the spheroidized powder as a reference state, the reflections of the lamellar cementite are (i) systematically shifted to lower 2*θ* angles and (ii) exhibit pronounced broadening. These phenomena were first quantified by pseudo-Voigt fits of individual reflections, and the resulting parameters—reflection position and full widths at half maximum (FWHM), as well as calculated interplanar spacing—are summarized for several reflections in Table 1. The last column of Table 1 lists the relative changes in lattice spacing of selected (*hkl*) crystallographic planes in spheroidized cementite ($d_{hkl}^{S}$), compared with the lamellar state ($d_{hkl}^{L}$), while Table 2 reports the Rietveld refinement obtained unit cell parameters for both morphologies. Together, Table 1 and Table 2 demonstrate systematically larger lattice parameters in lamellar cementite, which may be, at this stage, attributed to differences in cementite composition and/or to the distinct morphology of the cementite particles, as discussed below.

The observed reflection broadening (cf. Figure 3) was quantified by diffraction-profile modeling in TOPAS, identifying variations in the sizes of coherently diffracting domains as the primary broadening mechanism in both powders. Anisotropic microstrain broadening, evaluated using the Stephens model, accounts for only a minor fraction of the total broadening. For the spheroidized cementite powder, model refinement with a spherical domain shape yielded a volume-weighted average diameter of ~300 nm. In contrast, lamellar cementite, described by an anisotropic cuboidal domain model, exhibited volume-weighted dimensions of approximately 69 × 31 × 59 nm^3^, with the smallest dimension corresponding to the thickness of the cementite lamellae.

The close correspondence between SEM-determined particle sizes and the refined coherent diffraction length in spheroidized cementite implies that these particles are largely single-crystal-like. On the other hand, coherently diffracting domains in lamellar cementite are considerably smaller, indicating substantial partitioning of the lamellae into numerous coherently diffracting subdomains.

TEM-EDS analysis was performed in order to investigate the influence of the elemental composition of cementite particles on the unit cell parameters of the orthorhombic lattice (cf. Table 2). The results revealed a clear morphology-dependent partitioning of Cr (cf. Figures S5 and S6). Spheroidized cementite contains approximately 12 wt.% Cr, 82 wt.% Fe, and 6 wt.% C, whereas lamellar cementite contains about 2 wt.% Cr, 92 wt.% Fe, and 6 wt.% C. Within the typical 4–8% uncertainty of TEM-EDS quantification [29], these measurements indicate a higher concentration of substitutional Cr in the spheroidized particles. First principles studies of cementite type carbides have established that increasing Cr-substitution on the metallic sublattice leads to a systematic expansion of the cementite unit cell [18]. On purely compositional grounds, the higher Cr content in spheroidized cementite would be expected to result in larger lattice parameters than in the lamellar state. The diffraction data, however, show the opposite trend: the unit cell of lamellar cementite is expanded compared to the more Cr-rich spheroidized cementite (cf. Figure 3, Table 2). This inversion indicates that the measured differences between $d_{hkl}^{S}$ and $d_{hkl}^{L}$ (Table 1) cannot be explained by Cr partitioning alone and instead point toward a microstructural origin.

**Figure 4:**
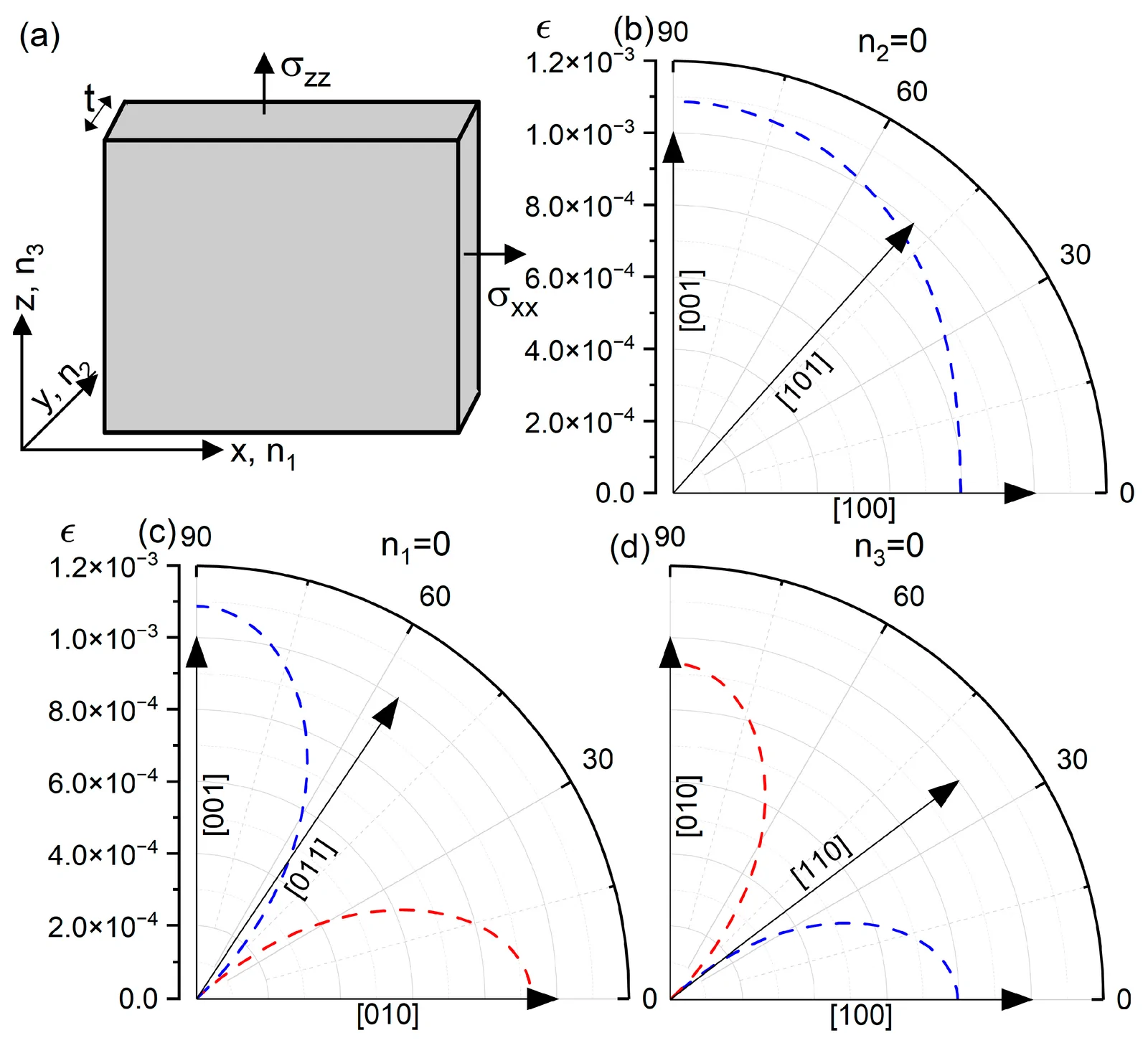
In (**a**), geometric representation of the lamellar particle model with a corresponding coordinate system is presented. Figures (**b**–**d**) show pole-figure projections in the (010) n_2_ = 0, (100) n_1_ = 0 and (001) n_3_ = 0 planes of orientation-dependent surface-induced strain. Reference crystallographic directions are indicated by arrows. Dashed lines represent the strain amplitude as a function of direction for lamellar cementite. The color scheme denotes the sign of the strain, with blue and red symbolizing tensile and compressive strain, respectively. For instance, (**b**) indicates that the relative change in *d_hkl_* of ~1.1 × 10^−3^ is maximal along the [001] direction and ~0.8 × 10^−3^ along the [100] direction. In contrast, strain projections in (**c**,**d**) show both positive and negative strain components, reflecting the non-hydrostatic nature of the surface-induced stress state associated with the lamellar geometry.

A continuum mechanics framework was established to interpret these discrepancies as direction-dependent elastic strains generated by surface stresses at free particle surfaces and elastically transmitted throughout the particle volume [30]. The model explicitly incorporates the low-symmetry orthorhombic crystal structure of cementite (Pnma) via its full elastic stiffness tensor [31]. Surface energy was taken as a positive, isotropic and scalar quantity. Under this assumption, the strain dependence of the surface energy (Shuttleworth effect [32]) vanishes, so that the surface energy and surface stress become equivalent. Spheroidized particles were treated as perfect spheres under purely hydrostatic Laplace pressure [33], whereas lamellar particles were analyzed in the thin-film limit [34] appropriate for the experimentally relevant lamella thickness of 10–30 nm, resulting in a non-hydrostatic stress state with a pronounced deviatoric component. Using the Bagaryatskii orientation relationship, the lamella axes schematically shown in Figure 4a were aligned with the principal crystallographic directions of cementite [35,36].

For a given *hkl* reflection, the relative change in interplanar spacing, $\left(d_{hkl}^{Q_{hkl}}-d_{0}\right)/d_{0}$, is obtained by projecting the elastic strain tensor *ε_ij_* along the corresponding diffraction vector *Q_hkl_*. On this basis, closed-form expressions for the reflection-specific changes in *d_hkl_* were derived for spherical (Equation (1)) and lamellar particles (Equation (2)). Detailed derivation of the following relations is presented in the Supplementary Materials.

(1) $$\frac{d_{hkl}^{Q_{hkl}}-d_{0}}{d_{0}}=\varepsilon_{ij}n_{i}n_{j}\;=-2\gamma \left(n_{1}^{2}\left(S_{11}+S_{12}+S_{13}\right)+n_{2}^{2}\left(S_{12}+S_{22}+S_{23}\right)+n_{3}^{2}\left(S_{13}+S_{23}+S_{33}\right)\right)exp\left(-\mu -0.5\left(2q-1\right)\sigma^{2}\right)$$

(2) $$\frac{d_{hkl}^{Q_{hkl}}-d_{0}}{d_{0}}=\varepsilon_{ij}n_{i}n_{j}=2\gamma /t\left(n_{1}^{2}\left(S_{11}+S_{13}\right)+n_{2}^{2}\left(S_{12}+S_{23}\right)+n_{3}^{2}\left(S_{13}+S_{33}\right)\right);\;\sigma_{yy}=0$$

where *d*_0_ is the interplanar spacing of a reference, unstrained standard, *n_i_* represents normalized direction cosine vector components of *Q_hkl_*, *γ* [J/m^2^] is the surface energy, and *S_ij_* are components of the elastic compliance tensor [GPa^−1^] in the Voigt notation. The last term in Equation (1) accounts for the lognormal particle-diameter distribution; *q* is the weighting factor, *μ* the mean and *σ* the standard deviation. In Equation (2), the change in lattice spacing depends on the same elastic properties but scales with lamella thickness *t* and is formulated for the σ*_yy_* = 0 plane-stress condition. Equivalent expressions for lamellar particles for σ*_xx_* = 0 and σ*_zz_* = 0 plane-stress conditions are presented in Supplementary Materials.

The changes in ($d_{hkl}^{L}$ − $d_{hkl}^{S}$)/$d_{hkl}^{L}$ predicted by Equation (2) for various orientations of the diffraction vector *Q_hkl_* with respect to the cementite lamellar vectors *x*, *y* and *z*, where *z* is perpendicular to the lamella surface, are shown in polar coordinates in Figure 4.

Pole-figure projections of the surface-induced strain for the cases of σ*_xx_* = 0 and σ*_zz_* = 0 are presented in Figure S7. The values of *S_ij_* were taken from Ref. [37], and γ = 2.2 Jm^−2^, corresponding to the orientation-averaged DFT surface energy of low-index cementite facets [38,39]. The adopted surface energy therefore represents an approximation, reflecting both the uncertainty of the first-principles prediction and the use of an orientation-averaged value for a crystallographically anisotropic property. Consequently, the predicted strain magnitude scales linearly with the adopted value of *γ*.

The surface-induced strain projections presented in Figure 4 and Figure S7 were calculated using a lamella thickness of 20 nm, as determined from TEM microstructural analysis shown in Figure S6. Strains in lamellar cementite were evaluated relative to the refined lattice parameters of spheroidized cementite, treated as an effectively unstrained reference state due to its order of magnitude larger characteristic particle size. The projections in Figure 4b–d show that positive surface energy *γ* produces in-plane expansion of the lamella and out-of-plane contraction. These mixed anisotropic lattice parameter changes, arising from Poisson coupling [40], are manifested as blue (expansion) and red (compressive) sectors in Figure 4, which map the sign of the strain and its variation along [*h*0*l*], [0*kl*] and [*hk*0] directions. Consequently, diffraction vectors sampling different crystallographic directions probe varying combinations of positive and negative strain components, yielding projected lattice strains of opposite signs depending on lamella orientation. This behavior underscores the strong sensitivity of diffraction-measurable strain to lamella orientation in the diffraction geometry.

In the powder-diffraction experiments, an ensemble of randomly oriented crystallites is sampled, so the measured change in *d_hkl_* represents an orientation-averaged lattice parameter along the diffraction vector *Q_hkl_*. Powder-averaged surface-induced lattice strains, corresponding to an average volumetric strain, for spherical and lamellar cementite were obtained by angular averaging of Equations (1) and (2), yielding Equations (3) and (4), respectively:

(3) $$\langle \varepsilon_{powder}^{S}\rangle =-1/32\gamma \left(\left(S_{11}+S_{12}+S_{13}\right)+\left(S_{12}+S_{22}+S_{23}\right)+\left(S_{13}+S_{23}+S_{33}\right)\right)exp\left(-\mu -0.5\left(2q-1\right)\sigma^{2}\right)$$

(4) $$\langle \varepsilon_{powder}^{L}\rangle =2\gamma /t\;1/3\left(\left(S_{11}+S_{13}\right)+\left(S_{12}+S_{23}\right)+\left(S_{13}+S_{33}\right)\right);\;\sigma_{yy}=0$$

Within this framework, Equation (4) predicts a relative change in *d_hkl_* = 3.5 × 10^−4^ for lamellar cementite, evaluated with respect to spheroidized cementite as the reference state, at lamellae thickness t = 20 nm. Under the condition of σ*_xx_* = 0 and σ*_zz_* = 0, the corresponding equations predict the relative change in *d_hkl_* of 3.0 × 10^−4^ and 3.2 × 10^−4^, respectively. These values represent roughly half the mean shift in experimentally observed average volumetric strain of 6.3 ± 2.2 × 10^−4^, reported in Table 1.

The discrepancy between the experimentally determined and model-predicted lattice strain is expected to arise from limitations of the modeling framework rather than a single physical origin. First, the continuum mechanics framework considers only the elastic strain generated by surface stress and neglects additional lattice distortions associated with crystallographic defects. Both line defects (dislocations) and point defects can introduce local elastic strain fields that contribute to the experimentally measured lattice strain but are not incorporated in the present framework. Second, the crystallographic direction averaging employed in Equation (4) inherently reduces the anisotropic strain state of orthorhombic cementite to a single powder-averaged value. This simplification is expected to constitute the dominant source of the observed discrepancy. The model further assumes an isotropic, scalar surface energy, whereas first-principal calculations have demonstrated that the surface energy of orthorhombic cementite exhibits a crystallographic orientation dependence. Consequently, the predicted strain represents an average approximation rather than the reflection-specific strain state that can be calculated from Equation (2) for the lamellar morphology. This is consistent with the experimental observations summarized in Table 2, where the (121), (031), and (230) reflections exhibit substantially larger lattice strain than the remaining reflections, thereby increasing the experimentally determined average lattice strain. Such reflection-dependent behavior cannot be reproduced within the present isotropic surface-energy approximation. Furthermore, minor differences in cementite composition, particularly in Cr content between the lamellar and spheroidized microstructures indicated by TEM-EDS, may additionally influence the lattice parameters; however, this contribution is expected to be secondary compared with the effects of crystallographic defects and the simplified treatment of the anisotropic surface stress.

Nonetheless, both the sign (positive) and the magnitude of the predicted lattice-parameter increase agree well with the observed shift in lamellar reflections to lower 2*θ*. This agreement supports free-surface-induced stress as a valid contributor to the mean reflection displacement.

## 4. Conclusions

Cementite morphology is shown to induce a measurable mean lattice strain in nanostructured cementite powders, with lamellar cementite exhibiting larger lattice parameters than spheroidized cementite despite its lower Cr content. Powder-diffraction and TEM-EDS data, combined with DFT trends for Cr-substituted cementite, demonstrate that composition alone cannot account for the observed average changes in $d_{hkl}^{L}$ by ~0.06%, compared to the $d_{hkl}^{S}$ (Table 1), implicating morphology-dependent internal stress instead. A surface-stress-based continuum-mechanics model that incorporates cementite’s orthorhombic elastic anisotropy quantitatively links positive surface energy to in-plane expansion and out-of-plane contraction in lamellae and predicts a powder-averaged lattice strain of 3.2 × 10^−4^, in reasonable agreement with the diffraction-derived mean shifts. Morphology-induced surface strain thus constitutes a systematic bias in diffraction-derived lattice parameters of nanostructured carbides and, more generally, of nanostructured particles with diverse morphologies, with direct consequences for diffraction-based residual-stress and strain analysis in engineering alloys. Although the presented model captures the dominant morphology-dependent strain contributions, future work incorporating the influence of point and line defects, anisotropic surface energy, or other atomistic effects may further refine the description of surface-induced lattice strain in complex geometries.

## Figures and Tables

**Table 1:** Diffraction peak parameters of selected reflections for lamellar and spheroidized particles including Miller indices (*hkl*), reflection position 2*θ*, full widths at half maximum (FWHM), lattice plane spacing *d*, and the calculated relative shift in the *d*-spacing between the two morphologies.

			Lamellar			Spherical			
*h*	*k*	*l*	2*θ* [deg]	FWHM [deg]	$d_{hkl}^{L}$ [Å]	2*θ* [deg]	FWHM [deg]	$d_{hkl}^{S}$ [Å]	$\frac{d_{hkl}^{L}-d_{hkl}^{S}}{d_{hkl}^{L}}$ × 10^−4^
2	0	0	8.000	0.020	2.540	8.002	0.010	2.539	2.5
1	2	1	8.517	0.030	2.386	8.525	0.008	2.384	9.4
2	1	0	8.550	0.029	2.377	8.554	0.009	2.375	4.7
0	0	2	8.984	0.024	2.262	8.990	0.008	2.260	6.7
2	0	1	9.176	0.020	2.215	9.180	0.009	2.214	4.3
2	1	1	9.661	0.028	2.104	9.665	0.009	2.103	4.1
1	0	2	9.837	0.025	2.066	9.843	0.009	2.065	6.1
2	2	0	10.023	0.038	2.028	10.029	0.008	2.027	6.0
0	3	1	10.104	0.040	2.012	10.115	0.009	2.010	10.9
1	1	2	10.291	0.030	1.975	10.297	0.008	1.974	5.8
1	3	1	10.867	0.041	1.871	10.881	0.008	1.869	1.3
2	2	1	10.987	0.034	1.851	10.994	0.008	1.849	6.4
1	2	2	11.546	0.036	1.761	11.554	0.008	1.760	6.9
2	3	0	12.086	0.047	1.683	12.098	0.008	1.681	9.9
2	1	2	12.415	0.031	1.638	12.422	0.010	1.637	5.6
3	0	1	12.830	0.026	1.586	12.833	0.011	1.585	2.3
3	1	1	13.181	0.034	1.544	13.184	0.015	1.543	2.3
									6.3 ± 2.2

**Table 2:** Rietveld refined lattice parameters of lamellar and spheroidized cementite particles determined from double-Voigt diffraction profile modeling.

Lattice Parameter	Lamellar	Spheroidized
a [Å]	5.0788 ± 0.5 × 10^−4^	5.0782 ± 0.1 × 10^−4^
b [Å]	6.7371 ± 0.9 × 10^−4^	6.7306 ± 0.1 × 10^−4^
c [Å]	4.5232 ± 0.5 × 10^−4^	4.5211 ± 0.8 × 10^−4^

## Data Availability

The original data including recorded XRD diffractograms and TOPAS .inp files presented in this study are openly available in Mendeley Data at https://doi.org/10.17632/7xcrjd6dxx.1.

## Supplementary Materials

The following supporting information can be downloaded at: https://www.mdpi.com/article/10.3390/ma19163431/s1. The following supporting information, including supplementary Figures, Tables, and detailed derivations of presented Equations, are available in Supplementary Materials. Figure S1: (a) histogram of relative frequency of equivalent circle diameter (ECD). The orange line represents the fitted lognormal distribution, distribution parameters μ and σ are also presented. In (b) relative frequency histogram of spherical particle aspect-ratios.; Figure S2: Recorded engineering stress-strain curves for (a) spheroidized and (b) lamellar morphology.; Figure S3: High-resolution powder X-ray diffraction pattern recorded at ID22 of the ESRF from cementite powder with a lamellar morphology; Figure S4: High-resolution powder X-ray diffraction pattern recorded at ID22 of the ESRF from cementite powder with spheroidized morphology.; Figure S5: Bright-field STEM micrograph of the spheroidized microstructure; Figure S6: Bright-field STEM micrograph of the lamellar microstructure with alternating cementite-ferrite lamellae.; Figure S7: Pole-figure projections of the surface-induced strain for cases of in-plane stress in the (a–c) xy-direction (σzz = 0), (d–f) xz-direction (σyy = 0) and (g–i) yz-direction (σxx = 0); Table S1: Mechanical properties of tested specimens with the divorced eutectoid microstructure determined from the experimentally recorded engineering stress-strain curves; Table S2: Mechanical properties of tested specimens with the lamellar microstructure determined from the experimentally recorded engineering stress-strain curves.
