# Supplementary material for: Surface-Stress-Induced Lattice Distortion in Nanolamellar Cementite
Source: Materials (Basel). 2026 Aug 13;19(16):3431. doi: 10.3390/ma19163431 (PMC13514185; doi:10.3390/ma19163431)
Supplement: Supplementary file 1 [file materials-19-03431-s001.zip › materials-4426797-supplementary.pdf]

Supplementary Material

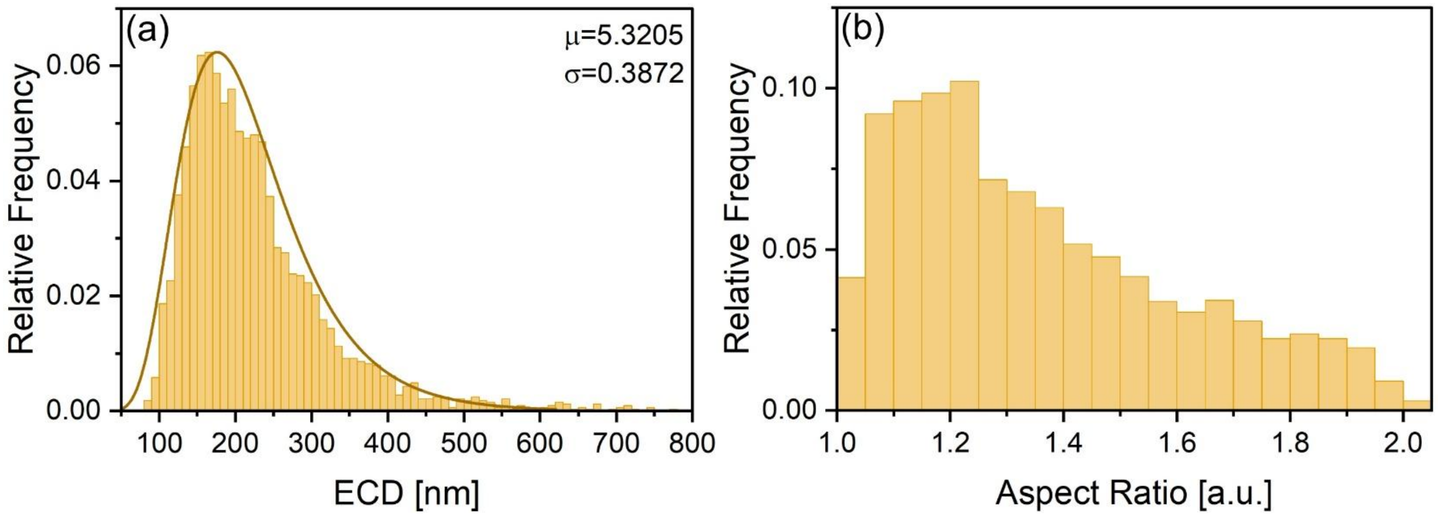

Fig. S1: (a) histogram of relative frequency of equivalent circle diameter (ECD). The orange line represents the fitted lognormal distribution, distribution parameters μ and σ are also presented. In (b) relative frequency histogram of spherical particle aspect-ratios.

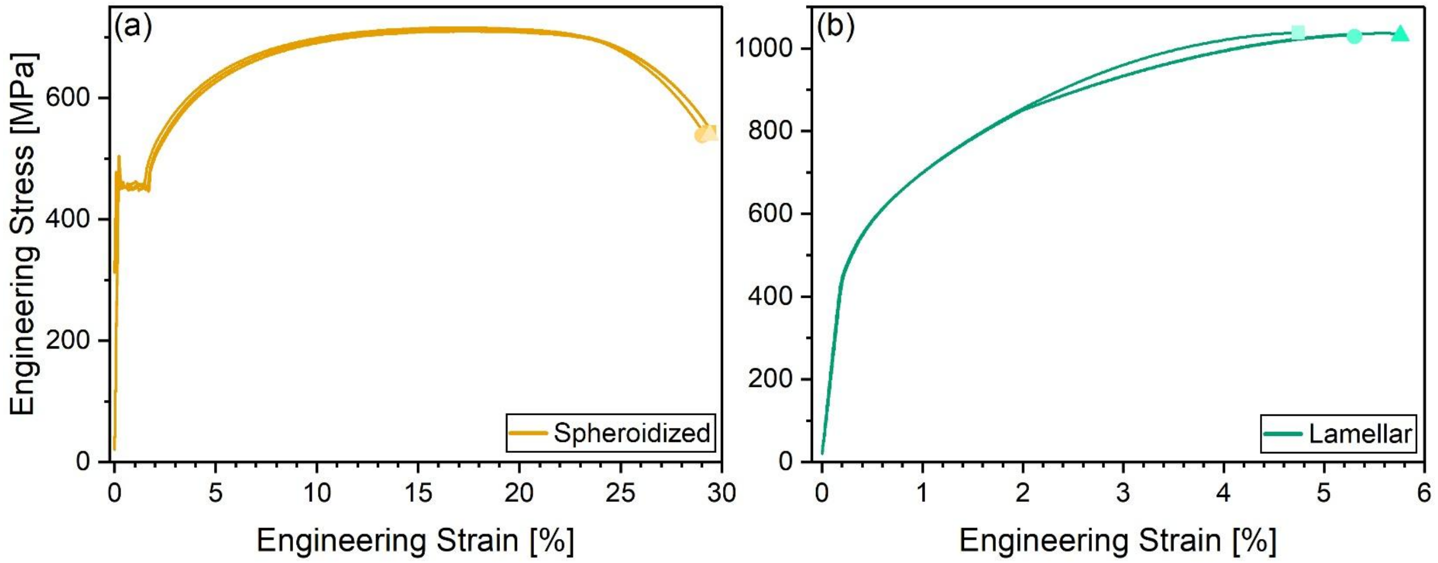

Fig. S2: Recorded engineering stress-strain curves for (a) spheroidized and (b) lamellar morphology. Geometric shapes mark the point of failure of the material.

Table S1: Mechanical properties of tested specimens with the divorced eutectoid microstructure determined from the experimentally recorded engineering stress-strain curves.

Divorced Eutectoid Microstructure	Max. Elongation [%]	UTS [MPa]	Young´s Modulus [GPa]	Lower Yield Strength [MPa]	Upper Yield Strength [MPa]	Yield Point Elongation [%]
Reference	29.4	710	221	446	481	1.52
Reference	29.0	716	220	450	479	1.5
Reference	29.4	713	228	451	505	1.45
**Average**	**29.3 ± 0.17**	**713 ± 2.0**	**223 ± 3.3**	-	-	**1.5 ± 0.03**

Supplementary Table S2: Mechanical properties of tested specimens with the lamellar microstructure determined from the experimentally recorded engineering stress-strain curves.

Lamellar Microstructure	Max. Elongation [%]	UTS [MPa]	Young´s Modulus [GPa]	$R_{p0.2}$ [MPa]
Reference	4.3	1038	214	567
Reference	4.9	1033	227	565
Reference	5.3	1037	224	566
**Average**	**4.8 ± 0.4**	**1036 ± 2.0**	**222 ± 5**	**566 ± 1**

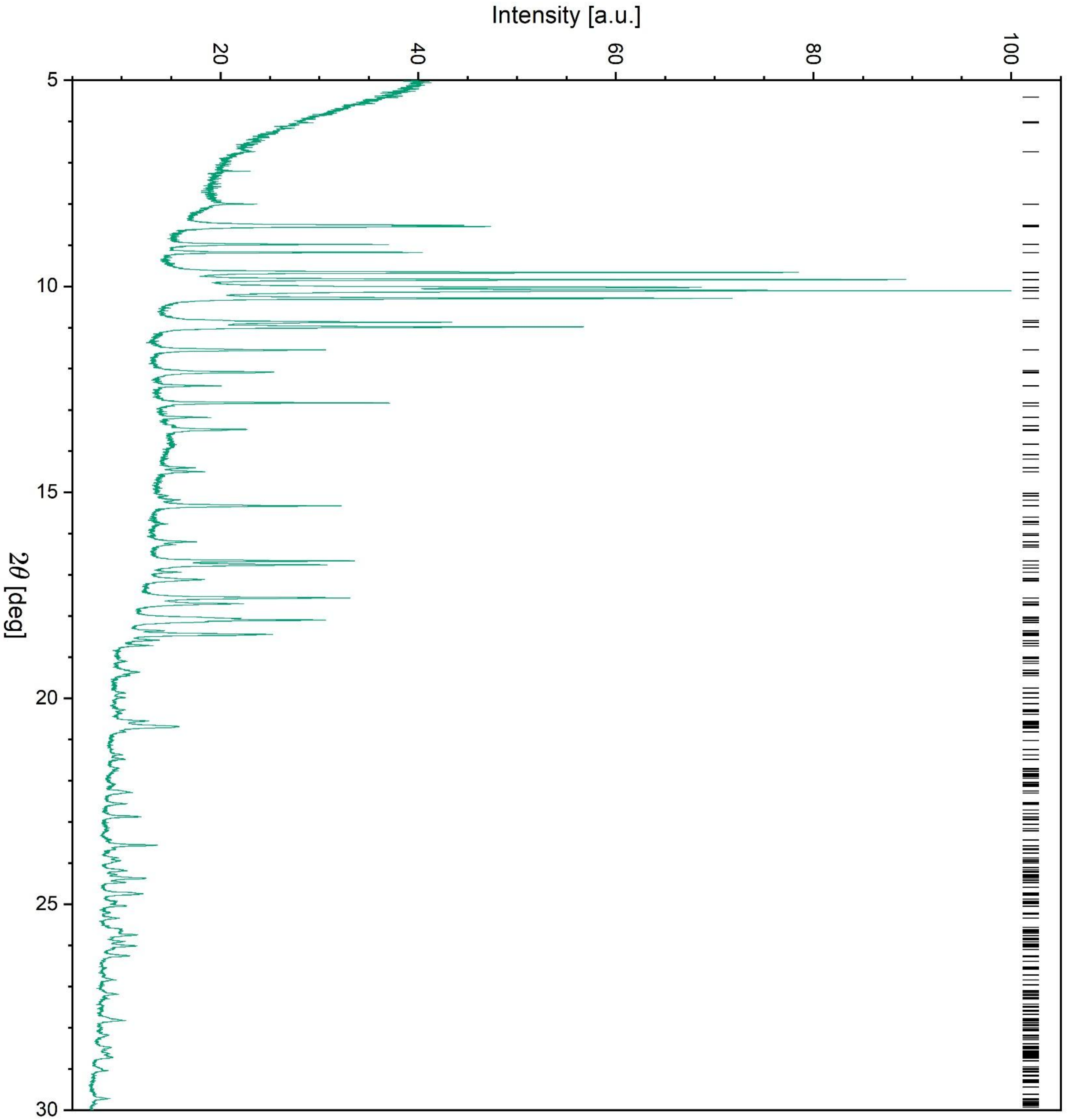

Fig. S3: High-resolution powder X-ray diffraction pattern recorded at ID22 of the ESRF from cementite powder with a lamellar morphology. Black lines mark the orthorhombic Fe_3C reflections.

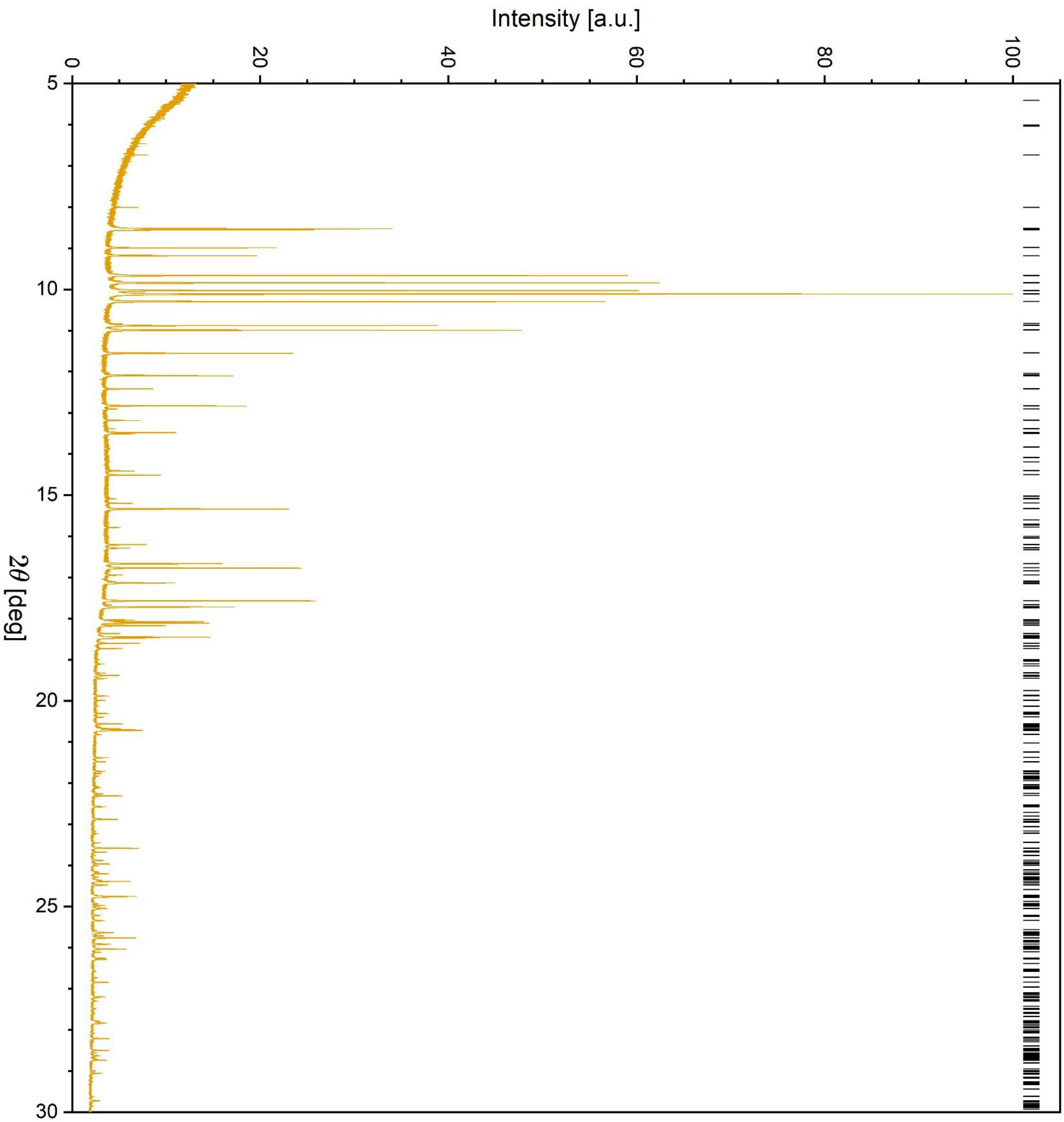

Fig. S4: High-resolution powder X-ray diffraction pattern recorded at ID22 of the ESRF from cementite powder with spheroidized morphology. Black lines mark the orthorhombic Fe_3C reflections.

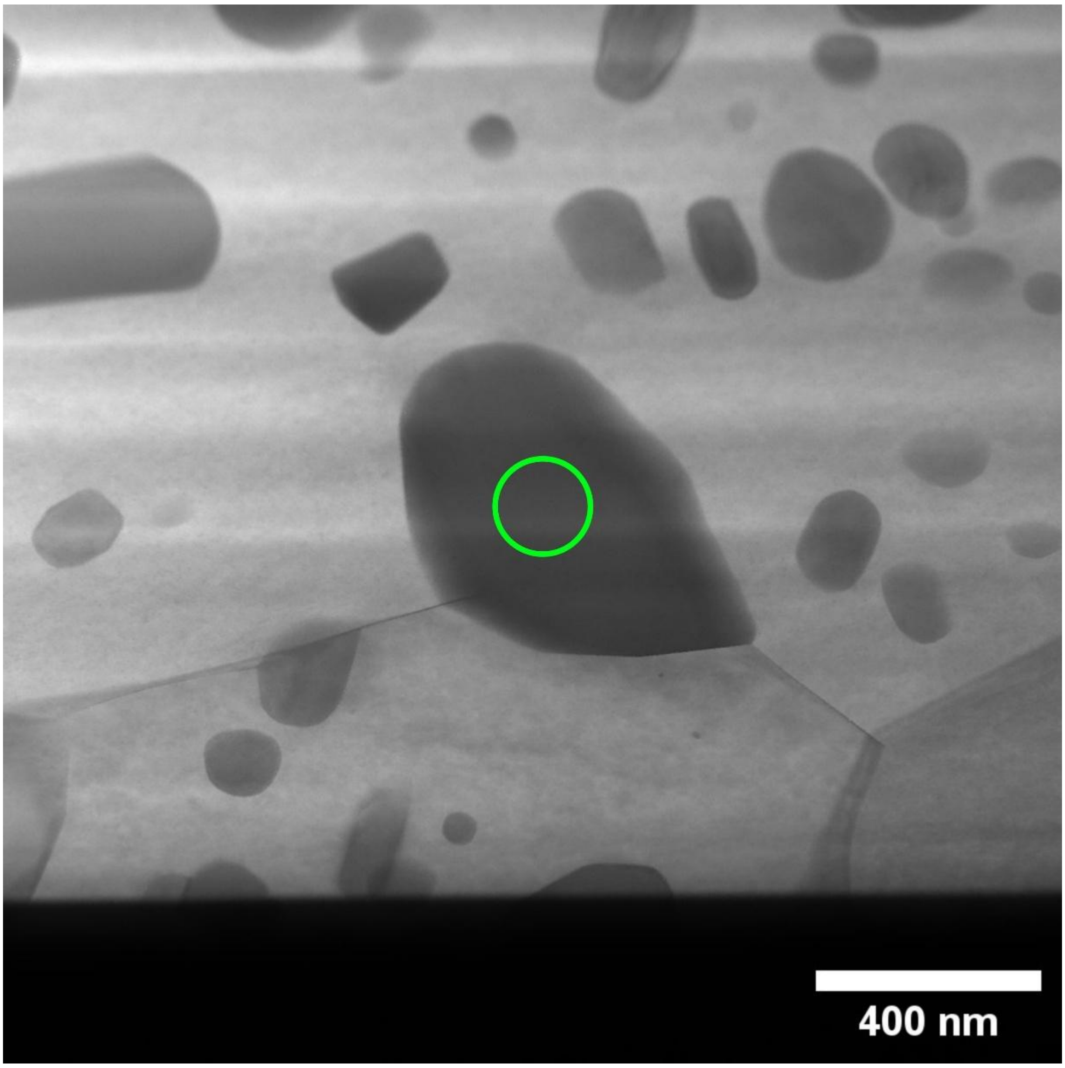

Fig S5: Bright-field STEM micrograph of the spheroidized microstructure. The green circle marks the region selected for TEM–EDS elemental mapping. The in-text reported values represent the average elemental wt.% inside the selected region.

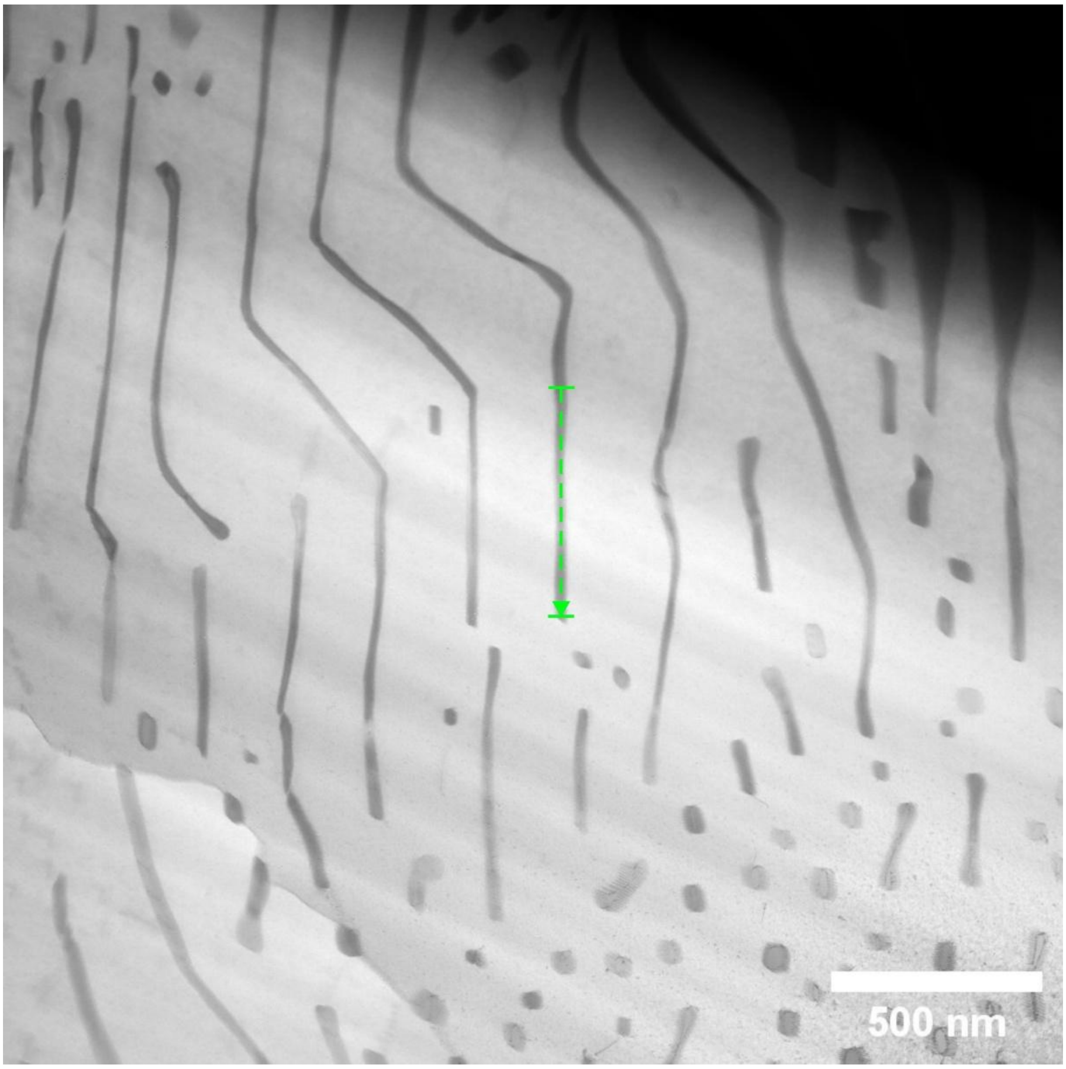

Fig S6: Bright-field STEM micrograph of the lamellar microstructure with alternating cementite-ferrite lamellae. The green line along one of the cementite lamellae marks the region selected for TEM–EDS elemental mapping. The in-text reported values represent the average elemental wt.% along this line.

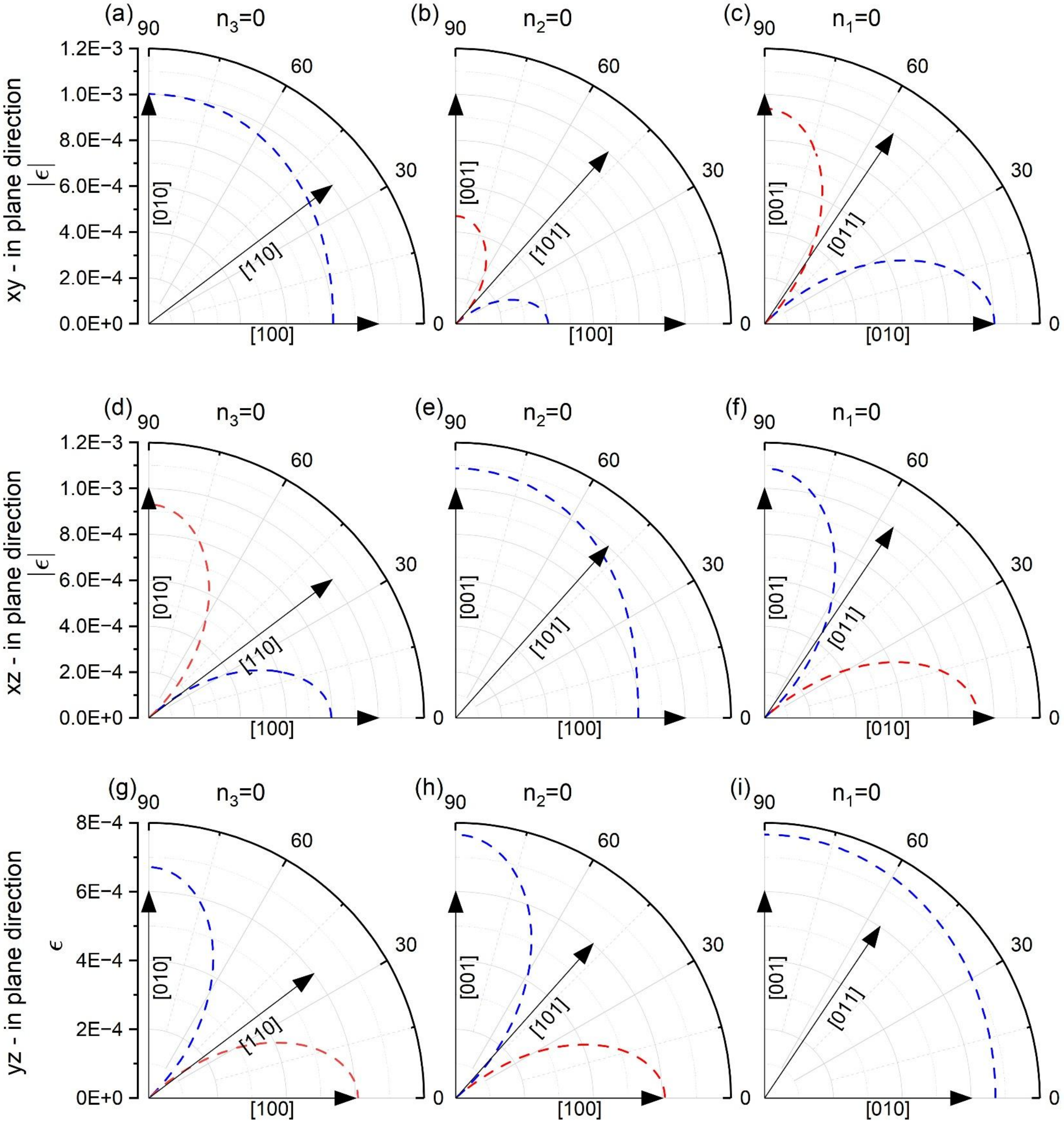

Fig. S7: Pole-figure projections of the surface-induced strain for cases of in-plane stress in the (a-c) xy-direction (σ_{zz}=0), (d-f) xz-direction (σ_{yy}=0) and (g-i) yz-direction (σ_{xx}=0). Reference crystallographic directions are indicated by arrows. Dashed lines represent the strain amplitude as a function of direction for lamellar cementite. The color scale denotes the sign of the strain, with blue and red symbolizing tensile and compressive strain, respectively.

1. Supplementary Surface-Induced Stress – General Assumptions

Both morphologies were treated within a linear-elastic continuum framework, modelling cementite as an orthorhombic solid (Pnma) described by the full elastic tensor. Surface-induced stresses were assumed to be generated at free particle surfaces and transmitted elastically through the particle volume, yielding a uniform internal stress state determined solely by particle geometry. Throughout, the surface energy was taken to be scalar and isotropic and the Shuttleworth effect was neglected such that surface stress was equated to surface energy. This assumption is supported by previous DFT simulations [37] and experimental observation of low aspect-ratios of spheroidized particles. Furthermore, the full orthorhombic elastic stiffness tensor was considered. Individual values obtained from Ref. [35] and elastic compliance tensor values calculated by matrix inversion. Full elastic stiffness and compliance tensors in Voigt notation are shown below:

$$C_{ij} = \begin{pmatrix} 315 & 136 & 131 & 0 & 0 & 0 \\ 136 & 321 & 175 & 0 & 0 & 0 \\ 131 & 175 & 299 & 0 & 0 & 0 \\ 0 & 0 & 0 & 24 & 0 & 0 \\ 0 & 0 & 0 & 0 & 142 & 0 \\ 0 & 0 & 0 & 0 & 0 & 138 \end{pmatrix}$$

$$S_{ij} = 10^{-3} \begin{pmatrix} 4.14075 & -1.12392 & -1.15636 & 0 & 0 & 0 \\ -1.12392 & 4.88015 & -2.36385 & 0 & 0 & 0 \\ -1.15636 & -2.36385 & 5.23464 & 0 & 0 & 0 \\ 0 & 0 & 0 & 4.16667 & 0 & 0 \\ 0 & 0 & 0 & 0 & 7.04225 & 0 \\ 0 & 0 & 0 & 0 & 0 & 7.24638 \end{pmatrix}$$

C_{ij} – Elastic stiffness tensor with elastic moduli (GPa) under zero pressure at 0 K obtained from first principle calculations. S_{ij} – Elastic stiffness tensor with elastic compliance moduli (GPa^{-1}) calculated from C_{ij} by matrix inversion.

1.1. Supplementary Derivation of the Surface-Induced Strain in Spherical Particles

Spheroidized cementite particles were modelled as perfect spheres, characterized by a radius *R* with a free surface characterized by an isotropic, scalar surface energy γ.

For a surface with principal curvatures $\kappa_1=1/R_1$ and $\kappa_2=1/R_2$, mechanical equilibrium across a surface with isotropic surface energy is governed by the Young-Laplace relation:

$$p = \gamma(\kappa_1 + \kappa_2) = 2\gamma/R$$

p – Laplace pressure [Pa], γ – surface energy [J/m²], κ – curvature, R – mean particle radius

The surface-induced pressure acts compressively on the particle interior and the internal stress state is therefore purely hydrostatic.

$$\sigma_{ii} = -p\delta_{ij} = -\,2\gamma/R$$

$$\sigma_{\mathrm{xx}} = \sigma_{\mathrm{yy}} = \sigma_{\mathrm{zz}} = -\,2\gamma/R = \sigma_0, \sigma_{\mathrm{ij}} = 0\ (i \neq j)$$

σ_{ij} – elastic stress tensor, δ_{ij} – Kronecker delta

In Voigt notation, the stress tensor can be expressed as:

$$\sigma = (\sigma_{xx}, \sigma_{yy}, \sigma_{zz}, \sigma_{yz}, \sigma_{xz}, \sigma_{xy})^{\mathrm{T}} = (\sigma_0, \sigma_0, \sigma_0, 0, 0, 0)^{\mathrm{T}}$$

For a general linear elastic solid, Hooke´s law defines the relationship between stress and strain through the elastic stiffness tensor:

$$\sigma_{ij} = C_{ijkl}\varepsilon_{kl} \tag{S1}$$

ε_{ij} – elastic strain tensor, C_{ijkl} – elastic stiffness tensor, σ_{kl} – elastic stress tensor

The elastic strain follows from Equation S1:

$$\varepsilon_{ij=} S_{ijkl}\, \sigma_{kl} = -p\, S_{ijkk}\, \sigma_{kk} \tag{S2}$$

ε_{ij} – elastic strain tensor, S_{ijkl} – elastic compliance tensor, σ_{kl} – elastic stress tensor

In the Voigt notation, Equation S2 can be represented in vector form as:

$$\begin{pmatrix}\varepsilon_1\\ \varepsilon_2\\ \varepsilon_3\\ \varepsilon_4\\ \varepsilon_5\\ \varepsilon_6\end{pmatrix} = \begin{pmatrix} S_{11} & S_{12} & S_{13} & 0 & 0 & 0\\ S_{21} & S_{22} & S_{23} & 0 & 0 & 0\\ S_{31} & S_{32} & S_{33} & 0 & 0 & 0\\ 0 & 0 & 0 & S_{44} & 0 & 0\\ 0 & 0 & 0 & 0 & S_{55} & 0\\ 0 & 0 & 0 & 0 & 0 & S_{66}\end{pmatrix}\begin{pmatrix}-p\\ -p\\ -p\\ 0\\ 0\\ 0\end{pmatrix}$$

and the non-zero normal strain components:

$$\varepsilon_{\mathrm{xx}} = -2\,\gamma/R\,(S_{11} + S_{12} + S_{13})$$
$$\varepsilon_{\mathrm{yy}} = -2\,\gamma/R\,(S_{21} + S_{22} + S_{23})$$
$$\varepsilon_{\mathrm{zz}} = -2\,\gamma/R\,(S_{31} + S_{32} + S_{33})$$

The change in the interplanar spacing, lattice strain, measured by X-ray diffraction for a given *hkl* reflection $\left(d_{hkl}^{Q_{hkl}} - d_0\right)/d_0$ is obtained by projecting the strain tensor ε_{ij} along the corresponding diffraction vector $\boldsymbol{Q}_{hkl}$, where n_i represents the normalized direction cosine components of $\boldsymbol{Q}_{hkl}$ in the crystal frame.

$$\frac{d_{hkl}^{Q_{hkl}} - d_0}{d_0} = \varepsilon_{ij}n_in_j = n_1^2\varepsilon_1 + n_2^2\varepsilon_2 + n_3^2\varepsilon_3 + 2n_2n_3\varepsilon_4 + 2n_1n_3\varepsilon_5 + 2n_1n_2\varepsilon_6$$

$$= -2\,\gamma/R\,\left(n_1^2(S_{11} + S_{12} + S_{13}) + n_2^2(S_{12} + S_{22} + S_{23}) + n_3^2(S_{13} + S_{23} + S_{33})\right) \tag{S3}$$

Equation S3 explicitly depends on the particle radius R. In this model supported by experimental observations, particle radii are distributed according to the lognormal distribution function characterized by μ and σ. Performing the radius averaging with a weighing factor q = 3 (volume averaging) yields:

$$\frac{d_{hkl}^{Q_{hkl}} - d_0}{d_0} = \varepsilon_{ij}n_in_j$$
$$= -2\gamma\big(n_1^2(S_{11} + S_{12} + S_{13}) + n_2^2(S_{12} + S_{22} + S_{23})$$
$$+ n_3^2(S_{13} + S_{23} + S_{33})\big)\frac{\int \frac{1}{R}\, R^q \mathrm{w}(R)\mathrm{d}R}{\int R^q \mathrm{w}(R)\mathrm{dR}} =$$

$$= -\alpha(\gamma, \vec{n}) \frac{\mathrm{E}[\mathrm{R}^{q-1}]}{\mathrm{E}[\mathrm{R}^{q}]} = -\alpha(\gamma, \vec{n}) \exp(-\mu - 0.5(2q-1)\sigma^2) \quad \text{(S4)}$$

Equation S4 computes direction-dependent surface-induced strain in particles with spherical morphology and a lognormal distribution of the particle radii.

1.2. Supplementary Derivation Surface-Induced Strain in Lamellar Particles

Lamellar cementite particles were modelled as thin plates characterized by a thickness t, much smaller than their lateral dimensions. The particles were assumed to consist of two opposing large faces, approximately planar and parallel. The crystallographic and particle coordinate systems were assumed to coincide following the Bagaryatskii orientation relationship, which states that the most likely cementite habit planes on the ferrite-cementite interfaces are (010), (100) and (001) [35].

As the large lamellar faces are planar, curvature-induced Laplace pressure is negligible. Instead, surface effects enter through thickness-driven membrane stress, where free surfaces exert a tangential surface stress γ, which was taken as equal to surface energy. For a thin lamella with two opposing large faces, these surfaces generate an in-plane membrane force of magnitude 2γ. When distributed over the thickness t, the average in-plane stress is:

$$\sigma_0 = 2\gamma/t$$

In the thin-film limit, the stress normal to the lamellar faces is assumed 0, and the stress state corresponds to plane stress.

Depending on the orientation of the lamellar faces relative to the crystallographic axes, three equivalent in-plane stress configurations can be defined. For example, for an *xz* in-plane stress (thin direction along *y*), the stress tensor in Voigt notation can be defined as:

$$\sigma = (\sigma_{xx}, \sigma_{yy}, \sigma_{zz}, \sigma_{yz}, \sigma_{xz}, \sigma_{xy})^T = (\sigma_0, 0, \sigma_0, 0, 0, 0)^T$$

This stress state is non-hydrostatic and contains a significant deviatoric component. The elastic strain tensor is obtained from previously defined Equation S2, yielding normal strain components for the *xz* in-plane stress configuration:

$$\varepsilon_{\mathrm{xx}} = 2\,\gamma/t\,(S_{11} + S_{13})$$
$$\varepsilon_{\mathrm{yy}} = 2\,\gamma/t\,(S_{21} + S_{23})$$
$$\varepsilon_{\mathrm{zz}} = 2\,\gamma/t\,(S_{31} + S_{33})$$

As in the previous case, the diffraction-measurable change in the interplanar spacing, lattice strain, for a given hkl reflection corresponds to the projection of the strain tensor along a diffraction vector $\boldsymbol{Q}_{hkl}$

$$\frac{d_{hkl}^{Q_{hkl}} - d_0}{d_0} = \varepsilon_{ij} n_i n_j = 2\,\gamma/t\left(n_1^2(S_{11} + S_{13}) + n_2^2(S_{12} + S_{23}) + n_3^2(S_{13} + S_{33})\right)$$

Equivalent expressions for the *xy* and *yz* in-plane configurations are obtained by selecting the corresponding non-zero stress components, $\sigma_{zz} = 0$ for the *xy* and σ_{xx} for the *yz* in-plane stress configuration.

Lamellar particles in powder form are randomly oriented with respect to the incident beam. The experimentally observed change in the interplanar spacing therefore

corresponds to an orientation-averaged projection of the strain tensor. Powder averaging is performed by averaging the directional cosines over all orientations:

$$\langle n_1^2 \rangle = \langle n_2^2 \rangle = \langle n_3^2 \rangle = 1/3$$

Applying the orientation averaging to the expression above yields the powder averaged lattice strain for lamellar particles

$\langle \varepsilon^{\mathrm{L}}_{\mathrm{powder}} \rangle = 1/3\ 2\gamma\big((S_{11} + S_{13}) + (S_{12} + S_{23}) + (S_{13} + S_{33})\big)$; $\sigma_{\mathrm{yy}} = 0$ $\langle \Delta \mathrm{d}/\mathrm{d}_0 \rangle_{\mathrm{powder}} =$ $\langle \varepsilon^{\mathrm{diff}} \rangle_{\mathrm{powder}} = 2\gamma/t\ 1/3\ (S_{11} + S_{13} + S_{21} + S_{23} + S_{13} + S_{33})$
